# Loss of myocardial protection against myocardial infarction in middle-aged transgenic mice overexpressing cardiac thioredoxin-1

**DOI:** 10.18632/oncotarget.7726

**Published:** 2016-02-25

**Authors:** Verónica D´Annunzio, Virginia Perez, Tamara Mazo, Marina C. Muñoz, Fernando P. Dominici, María C. Carreras, Juan José Poderoso, Junichi Sadoshima, Ricardo J. Gelpi

**Affiliations:** ^1^ Institute of Cardiovascular Physiopathology and Department of Pathology, Faculty of Medicine, University of Buenos Aires, Buenos Aires, Argentina; ^2^ Institute of Chemistry and Biological Physical Chemistry, School of Pharmacy and Biochemistry, University of Buenos Aires, Buenos Aires, Argentina; ^3^ Laboratory of Oxygen Metabolism, University Hospital, University of Buenos Aires, Buenos Aires, Argentina; ^4^ Department of Cell Biology and Molecular Medicine, Rutgers New Jersey Medical School, Newark, NJ, USA; ^5^ Member of the National Council of Scientific and Technological Research (CONICET), Buenos Aires, Argentina

**Keywords:** myocardial infarction, ischemia/reperfusion, thioredoxin-1, middle-aged, Gerotarget

## Abstract

Thioredoxin-1 (Trx1) protects the heart from ischemia/reperfusion (I/R) injury. Given that the age at which the first episode of coronary disease takes place has considerably decreased, life at middle-aged (MA) emerges as a new field of study. The aim was determine whether infarct size, Trx1 expression and activity, Akt and GSK-3β were altered in young (Y) and MA mice overexpressing cardiac Trx1, and in a dominant negative (DN-Trx1) mutant of Trx1. Langendorff-perfused hearts were subjected to 30 minutes of ischemia and 120 minutes of reperfusion (R). We used 3 and 12 month-old male of wild type (WT), Trx1, and DN-Trx1. Trx1 overexpression reduced infarct size in young mice (WT-Y: 46.8±4.1% *vs*. Trx1-Y: 27.6±3.5%, *p* < 0.05). Trx1 activity was reduced by 52.3±3.2% (*p* < 0.05) in Trx1-MA, accompanied by an increase in nitration by 17.5±0.9%, although Trx1 expression in transgenic mice was similar between young and middle-aged. The expression of p-Akt and p-GSK-3β increased during reperfusion in Trx1-Y. DN-Trx1 mice showed neither reduction in infarct size nor Akt and GSK-3β phosphorylation. Our data suggest that the lack of protection in Trx1 middle-aged mice even with normal Trx1 expression may be associated to decreased Trx1 activity, increased nitration and inhibition of p-Akt and p-GSK-3β.

## INTRODUCTION

Thioredoxin-1 (Trx1) is one of the most important cellular antioxidant systems known to date [1]. Particularly, Trx1 exerts an increase in lifespan and has a protective effect against ischemia/reperfusion (I/R) injury, reducing the infarct size [2-4]. In this sense, Nakamura *et al.* [5] showed in patients subjected to bypass surgery that thioredoxin inactivation was a deleterious mechanism in I/R injury. Similarly, Tao *et al.* [3] showed that administration of Trx1 *in vivo* exerts significant protective effects on myocardial apoptosis decreasing myocardial infarct size, by inhibiting p38-MAPK activation. Thus, it is clear that Trx1 has a protective effect against I/R injury. However, most of these studies were performed in young and healthy rodents [2-4].

It is also widely known that I/R injury is exacerbated in elderly populations and that many of the protective mechanisms lose their effect with advanced age [6, 7]. But it is not clear whether this also occurs in middle-aged, when the deleterious effects of aging are already taking place [8, 9]. This lack of studies in middle-aged is striking, since ischemic episodes in patients begins at that stage of life, and they are not exclusive of advanced age [8, 9]. There are numerous experimental evidence showing that mice older than 18 months exhibit a significant increment in reactive oxygen and nitrogen species (ROS and RNS) thus exacerbating I/R damage [6, 7]. Regarding Trx1 and aging, it has been demonstrated that infarct size and apoptosis increase in older animals due to thioredoxin physiological inactivation [10, 11]. However, it is known that even though oxidation processes start when life begins; it is in middle-aged that they reach sufficient levels to trigger deleterious mechanisms on different cell components [12], and this ROS increases is able to modify expression and/or activity of several proteins [13-15]. However if Trx1, at this stage of life, suffers alterations in its expression and/or activity has not been studied, neither have modifications in the infarct size behavior.

It has been also widely demonstrated that the activation of the PI3K/Akt complex triggers intracellular events, such as the inactivation of glycogen synthase kinase 3β (GSK-3β) [16]. This is a consequence of its phosphorylation by Akt, which confers protection against I/R damage, decreasing the infarct size [16-18]. Adluri *et al.* [19] showed that Trx1 overexpression induces Akt-signaling pathway compared to wild type mice during ischemic stress, and this could be related to a reduction in oxidative stress. Regarding GSK-3β and Trx1, Schenkel *et al.* [20] report that in later stages there is a decrease of Trx1 in parallel with some signaling proteins, including GSK-3β activation that is involved in maladaptative cardiac remodeling and ventricular dysfunction. For these reasons, it would be interesting to study the activation/inactivation of Akt and GSK-3β in an acute I/R protocol with overexpression of Trx1, and to compare the results between young and middle-aged mice.

Thus, the first objective of our work was to evaluate infarct size and ventricular function in young and also in middle-aged transgenic (TG) mice overexpressing Trx1. To further study the role of Trx1 in cardioprotection, we also use a dominant negative (DN-Trx1) mutant (C32S/C35S) of Trx1, with cardiac overexpression and inactivation of Trx1. Since Trx1 presents a decrease in activity and protein nitration in older mice, a second objective was to determine if middle-aged TG mice overexpressing Trx1 also present an increase in these inactivation mechanisms. Finally, a third objective was to examine if the protection conferred by Trx1 involves Akt and GSK-3β inhibition/phosphorylation.

## RESULTS

Table 1 shows the systolic behavior throughout the left ventricular developed pressure (LVDP, mmHg) and the maximal rate of rise of left ventricular pressure (LV+dP/dt_max_), at baseline and 30 minutes of reperfusion. In all groups, LVDP and LV+dP/dt_max_ were significantly lower compared to pre-ischemic values, but showed no significant differences among groups. Regarding myocardial stiffness, represented by the left ventricular end of diastolic pressure (LVEDP, mmHg), we found a significant increase at 30 minutes of reperfusion respective pre-ischemic values without changes among the groups.

**Table 1:** Left ventricular systolic and diastolic function

	Groups	Baseline	30min Rep
LVDP (mmHg)	Wt-Y	88.2 ± 2.3	27.9 ± 4.7*
	Wt-MA	80.5 ± 8.5	22.1 ± 7.8*
	Trx1-Y	87.7 ± 6.6	30.6 ± 3.5*
	Trx1-MA	87.1 ± 7.8	29.2 ± 7.7*
	DN-Trx1-Y	91.2 ± 5.4	32.4 ± 5.2*
	DN-Trx1-MA	92.7 ± 8.2	31.9 ± 4.7*
LVEDP (mmHg)	Wt-Y	7.8 ± 1.1	28.5 ± 5.8*
	Wt-MA	8.8 ± 2.8	30.2 ± 8.5*
	Trx1-Y	7.1 ± 0.8	21.4 ± 7.1*
	Trx1-MA	7.6 ± 1.1	23.3 ± 6.6*
	DN-Trx1-Y	8.2 ± 1.8	34.5 ± 4.2*
	DN-Trx1-MA	8.5 ± 0.9	29.4 ±3.9 *
LV+dP/dt_max_ (mmHg/sec)	Wt-Y	3151 ± 206	1285 ± 196*
	Wt-MA	2949 ± 217	1199 ± 257*
	Trx1-Y	2927 ± 301	1399 ± 128*
	Trx1-MA	3228 ± 339	1320 ± 239*
	DN-Trx1-Y	3308 ± 287	1289 ± 117*
	DN-Trx1-MA	3189 ± 294	1229 ±133*

LVDP: Left ventricular developed pressure; LVEDP: Left ventricular end diastolic pressure; LV+dP/dt_max:_ Left ventricular maximal rate of pressure increase. Rep: Reperfusion. *: p<0.05 vs respective baseline value. Wt: wild type; Trx1: transgenic mice thioredoxin-1 overexpression; DN-Trx1: Dominant negative for endogenous Trx1 in mice hearts Y: Young; MA: middle-aged.

**Figure 1:**
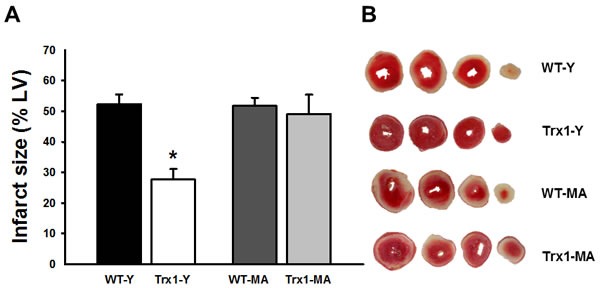
Infarct size expressed as a percentage of the total left ventricular area Infarct size decreased significantly in Trx1 young group but this cardioprotective effect was abolished in Trx1 middle-aged. Panel B shows representative slices of the different experimental groups. Data are represented as mean +/− SEM. *: *p* < 0.05 *vs*. WT-Y. WT-Y: wild type in young animals (3 month-old); WT-MA: wild type in middle-aged animals (12 month-old); Trx1-Y: thioredoxin-1 young group; Trx1-MA: thioredoxin-1 middle-aged group.

Figure 1 (Panel A) shows the infarct size in all experimental groups. The Trx1 overexpression significantly reduced the infarct size in young mice (WT-Y: 52.3±3.2% *vs*. Trx1-Y: 27.6±3.5%, *p* < 0.05), while middle-aged animals did not exhibit this infarct size reduction (WT-MA: 51.8±2.6% *vs*. Trx1-MA: 49.1±6.3%). Panel B shows representative slices of the different experimental groups.

**Figure 2:**
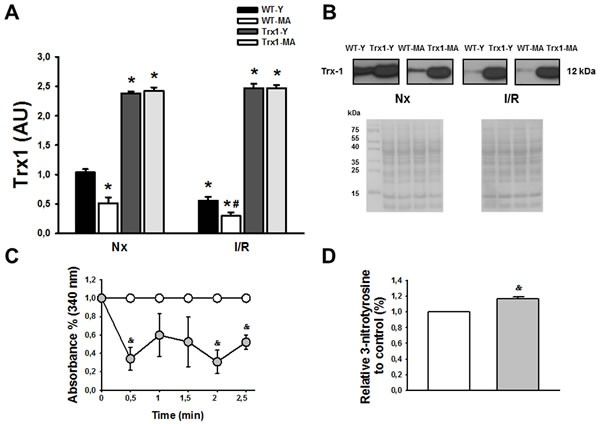
Panel A shows an increased in Trx1 expression in transgenic animals, both in young (Y) and middle-aged (MA) group in normoxic (Nx) conditions and after ischemia/reperfusion protocol The levels of Trx1 in wild type mice (WT) decreased at reperfusion (after 30 minutes of ischemia and 15 minutes of reperfusion) in young and middle-aged animals, but in transgenic mice the levels of Trx1 remains in similar values as normoxic conditions. Panel B show Ponceau S-stained blot, as loading control, and representative blots of Trx1. Panel C shows that in transgenic mice the activity was lower in Trx1 young group compared with Trx1 middle-aged group. Finally, in panel D we observed an increased in Trx1 nitration in middle-aged group compared with young group. Data are represented as mean +/− SEM. *: *p* < 0.05 *vs*. WT animals. #: *p* < 0.05 *vs*. Nx respective conditions; &: *p* < 0.05 *vs*. Trx1 young group.

Figure 2 shows the behavior of Trx1 expression (Panel A), activity (Panel C) and nitration (Panel D). In normoxic conditions in young and middle-aged TG mice there were a significant increase in Trx1 expression (Panel A), meanwhile Trx1 levels were lesser in WT middle-aged than in young mice (Nx-Y:1.1±0.1 *vs*. Nx-MA: 0.42±0.05, *p* < 0.05). At 15 minutes of reperfusion, Trx1 expression decreased significantly in wild type mice reaching a 47% in young and 42 % in middle-aged of reduction compared with pre-ischemic values (Panel A), and in TG mice Trx1 levels was similar to their respective normoxic values. Trx1 activity was reduced in Trx1-MA compared with Trx1-Y mice (Panel C). Directly related to the previous findings, there was a 17.5±0.9% increase in the nitration of Trx1-MA mice compared to Trx1-Y (Panel D). Taken together, these data suggest that in Trx1 middle-aged mice, even with normal Trx1 expression, there is inactivation of Trx1 that involves protein nitration. Panel B shows ponceau S-stained blot, as loading control, and representative blots of Trx1.

**Figure 3:**
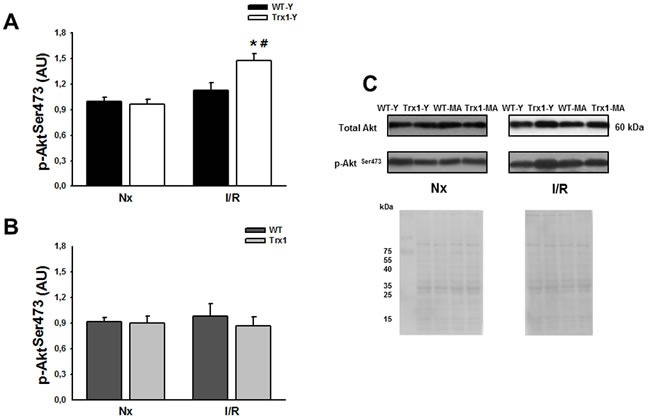
Akt phosphorylation (Ser 473) protein expression in the cytosolic fraction of normoxic (Nx) and ischemia/reperfusion protocols (I/R) in young (Panel A) and middle-aged (Panel B) mice There were not significantly changes in the cytosolic Akt protein expression (Panel C). Also, not significantly changes were detected, in the cytosolic p-Akt Ser 473 protein expression in Trx1 and WT middle-aged groups, however in Trx1 young group a significantly enhanced of Akt phosphorylation after I/R was detected. Panel C shows Ponceau S-stained blot, as loading control, and representative blots of total and phosphorylated Akt. Data are represented as mean +/− SEM. *: *p* < 0.05 *vs* normoxic Trx1; #: *p* < 0.05 *vs* WT-Y I/R. WT-Y: wild type in young animals (3 month-old); WT-MA: wild type in middle-aged animals (12 month-old); Trx1-Y: thioredoxin-1 young group; Trx1-MA: thioredoxin-1 middle-aged group.

Figure 3 shows the cardiac expression of Akt phosphorylation at Ser473 residue, in the cytosolic fraction in normoxic conditions (matched time) and after ischemia/reperfusion (15 minutes of reperfusion) in young (Panel A) and middle-aged mice (Panel B). Panel C shows representative blots and ponceau S-stained blot of total and phosphorylated Akt. We showed a significant increase in p-Akt Ser473 in the group of young animals with overexpression of Trx1 during reperfusion compared to pre-ischemic values (Trx1-Y; normoxic: 1.02±0.07 AU *vs*. I/R: 1.47±0.08 AU; *p* < 0.05), meanwhile in WT young group there were no changes at reperfusion period (Panel A). Nevertheless, p-Akt Ser473 values in middle-aged mice were similar between wild type and Trx1 before ischemia and during reperfusion (I/R: WT-MA 0.97±0.14 AU *vs*. Trx1-MA 0.86±0.10 AU) (Panel B). Thus, our data shows an increase in p-Akt Ser473 phosphorylation in Trx1-Y mice hearts while there is no increase in middle-aged ones. Regarding p-Akt Thr308 site (data not shown), there were no significant differences within the groups of young animals before ischemia and during reperfusion and middle-aged animals.

**Figure 4:**
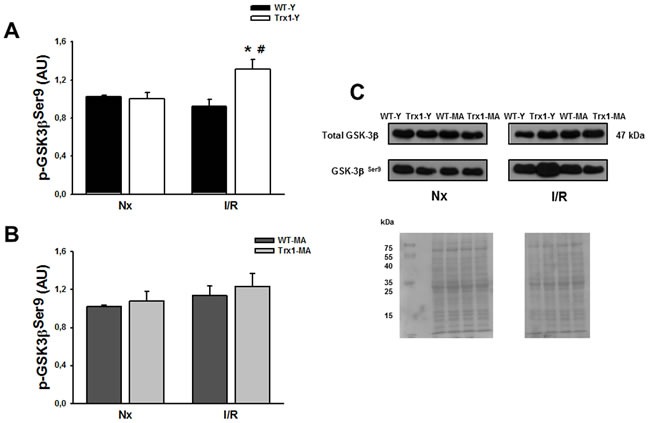
pGSK-3β Ser 9 protein expression in the cytosolic fraction of normoxic (Nx) and ischemia/reperfusion protocols (I/R) in young (Panel A) and middle-aged (Panel B) mice There were not significantly changes in the cytosolic GSK-3β protein expression (Panel C). Also, not significantly changes in the cytosolic in p-GSK-3β Ser 9 protein expression were detect in Trx1and WT middle-aged groups, however in Trx1 young group, a significantly enhanced of GSK-3β phosphorylation after I/R was detected. Panel C shows Ponceau S-stained blot, as loading control, and representative blots. Data are represented as mean +/− SEM. *:*p* < 0.05 *vs* normoxic Trx1; #: *p* < 0.05 *vs* WT-Y I/R. WT-Y: wild type in young animals (3 month-old); WT-MA: wild type in middle-aged animals (12 month-old); Trx1-Y: thioredoxin-1 young group; Trx1-MA: thioredoxin-1 middle-aged group.

Figure 4 shows the cardiac expression of p-GSK-3β Ser9 residue, in the cytosolic fraction in normoxic conditions and after ischemia/reperfusion (15 minutes of reperfusion in young (Panel A) and middle-aged mice (Panel B). It can be observed that there are no significant differences in the expression of total GSK-3β among all the groups studied (Panel C), while a significant increase of p-GSK-3β is observed in the group of young animals overexpressing Trx1 compared with its pre-ischemic value (Trx1-Y: normoxic: 1.01±0.01 AU *vs*. I/R: 1.30±0.10 AU, *p* < 0.05) (Panel A). However, this increase in the GSK-3β phosphorylation is not evidenced in the middle-aged groups, both in WT mice and in Trx1 mice (WT-MA: normoxic: 1.02±0.14 AU; I/R: 1.13±0.1 AU; Trx1-MA: normoxic: 1.08±0.09 AU; I/R: 1.23±0.13 AU) (Panel B). Panel C represents representative blots and ponceau S-stained blot of total and p-GSK-3β.

**Figure 5:**
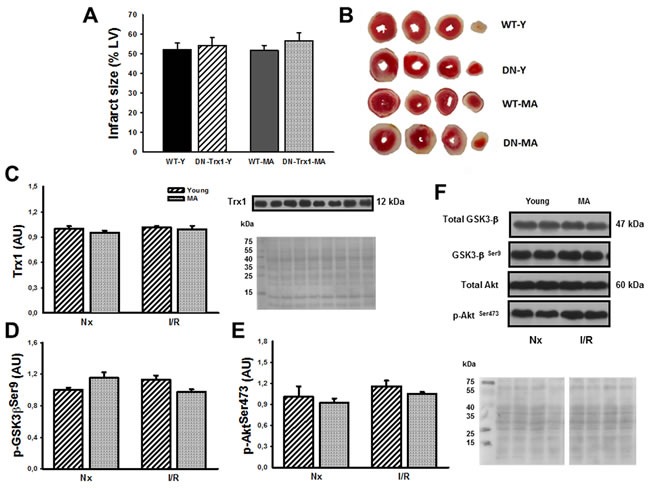
We showed DN-Trx1 mice results in order to confirm that the cardioprotection conferred by Trx1 involves Akt and GSK-3β inhibition/phosphorylation nfarct size (Panel A), Trx1 expression (Panel C), p-Akt Ser473 (Panel D) and p-GSK-3β Ser9 (Panel E) were similar between wild type (WT) and in dominant negative for Trx1 (DN-Trx1) in young and middle-aged mice. Panel B shows representative slide of infarct size. Panel F shows Ponceau S-stained blot, as loading control, and Akt/GSK-3β representative blots. Data are represented as mean +/− SEM.

In order to confirm that cardioprotection afforded by Trx1 involved Akt and GSK-3β phosphorylation, additional experiments in DN-Trx1 mice with cardiac overexpression of a redox inactive Trx1 mutant were performed. We detected that infarct size was similar between WT and DN-Trx1 in young (WT-Y: 52.3±3.2% *vs*. DN-Trx1-Y: 54.3±4.0%) and in middle-aged mice (WT-MA: 51.8±2.6% *vs*. DN-Trx1-MA: 56.6±4.2%). The Akt and GSK-3β phosphorylation in young and also in middle-aged DN-Trx1 did not show changes between basal and ischemia /reperfusion conditions. These results are consistent with the lack of cardioprotection in DN-Trx1 mice (Figure 5).

## DISCUSSION

The present study shows, as expected, that young mice overexpressing Trx1 have a smaller infarct size compared to their corresponding WT mice. However, this infarct size reduction was observed neither in Trx1 middle-aged nor in young and middle-aged DN-Trx1 mice. Although we found a significant increase in Trx1 levels in young and middle-aged transgenic mice, Trx1 activity was less in middle-aged mice and this findings was accompanied by an increase in the nitration of this protein. Finally, we also demonstrated that phosphorylation of Akt and GSK-3β were increased only in young Trx1 mice without any change in middle-aged mice and in DN-Trx1 mice.

Since we did not evidence infarct size reduction in middle-aged Trx1 mice, we mesaured Trx1 expression and activity in these animals. After ischemia/reperfusion protocol in WT mice we evidenced a significant reduction in Trx1 levels both in young and middle-aged animals. With regard to our findings, other authors have evidenced that during reperfusion after an ischemic period, there is an increase in peroxynitrite concentration, and it has been reported to produce cellular damage by lipid peroxidation, DNA fragmentation, and in addition induced depletion of antioxidants [21, 22] including thioredoxin [6]. However, in mice overexpressing thioredoxin we evidenced that during reperfusion Trx1 levels were similar to pre-ischemic values. Although protein levels remained intact, we found a decrease in Trx1 activity with an increase nitration, only in middle-aged mice. It is well known, that thioredoxin can suffer post-translational modifications such as S-nitrosylation, oxidation and nitration [23, 1, 10, 24]. The first one has beneficial effects improving Trx1 cardioprotective activity through apoptosis reduction [23]. Oxidation partially inactivates it while nitration causes Trx1 totally irreversible inactivation resulting in the attenuation of its biological functions [1, 10, 24]. Thus, it is clear that in our study the increase in Trx1 nitration produced the reduction in its activity and consequently there was no evidence of the beneficial effects on middle-aged mice infarct size. As previously mentioned, Zhang *et al.* [11] demonstrated that Trx activity is decreased only in the aging hearts of 20 month-old mice by post-translational nitrative modification. Interestingly our data shows that already at 12 month-old, when the deleterious effects of pro-oxidant mechanisms associated to aging are not completely established, are enough to detect and increase in Trx1 nitration and a consequent inactivation. It is probable that these post-translational modifications are related to the fact that no protection was evidenced in the size of the infarct. Neither were changes in the phosphorylation of Akt and GSK-3β in middle-aged mice.

Regarding Akt/p-Akt Ser473 in particular, Adluri *et al.* [19] demonstrated that Trx1 overexpression induces Akt-signaling pathway compared to WT mice during ischemic stress, and this could be related to a reduction in oxidative stress. These authors used a chronic model of myocardial infarction and showed that the Akt was phosphorylated several days after the ischemia insult. However, our research is the first that involves Akt as the probable target for thioredoxin to provide protection in an ischemia/reperfusion *in vitro* model in an acute way. This was supported by the fact that in DN-Trx1 mice phosphorylation of Akt and GSK-3β was not observed and therefore the infarct size was not reduced. Regarding interactions, it has been demonstrated that Trx1 is capable of indirectly phosphorylating Akt in other pathologies such as myocardial remodelling and cancer [21, 25, 26]. This protein has been extensively studied and has a major role in physiological and pharmacological protection mechanisms; it is also part of the RISK-pathway protection system [25, 27, 28]. A possible mechanism by which Trx relates to Akt is through PTEN, because it has been shown that Trx1 inhibits PTEN [29], as this mediator modulates activation of PI3K/Akt complex triggering intracellular events that confer protection against I/R injury [28]. A limitation to our study is that we did not evaluate the interaction between PTEN and Trx1, nevertheless when we used DN-Trx1mice we could not detect neither cardioprotection nor activation of pro-survival proteins. Therefore, our data suggest that overexpression of Trx1 protects the myocardium against ischemia/reperfusion injury by activating Akt and GSK-3β.

Our results confirm that the protection conferred by the overexpression of Trx1 would be at least partially due to inactivation of GSK-3β, given that in DN-Trx1 the lack of GSK-3β phosphorylation showed that this pro-survival protein was not activated [30]. To our knowledge, this is a novel finding since, no studies have assessed if Trx1 has an effect on GSK-3β in an acute way, once reperfusion is initiated, in young and middle-aged mice comparatively. Unlike our findings, Schenkel *et al.* [20] demonstrated that after myocardial infarction, even though Trx1 appears to make an important contribution to the reduced H_2_O_2_ concentration, is not enough to produce modifications in GSK-3β in the early stages in a chronic model without reperfusion. This difference is important; given that Zhai *et al.* [30] demonstrated that the inhibition of GSK-3β does not produce a cardioprotective effect, but exacerbates the ischemic injury in a mice model with ischemia without reperfusion. However, it does have a protective effect against ischemic injury followed by reperfusion through the modulation of autophagy [30]. Thus, in concordance with our findings, the inactivation of GSK-3β would have a protective effect in I/R models, like our experimental model. This inactivation of GSK-3β would have a beneficial effect in young mice overexpressing Trx1; meanwhile there is no evidence of phosphorylation or infarct reduction in middle-aged Trx1 and in DN-Trx1 mice.

In agreement with previous studies using isolated hearts mice preparations [31] in our study, overexpression of Trx1 in young mice reduced infarct size but was unable to improve ventricular function (contractile state and myocardial stiffness). The absence of improvement of LV function in our study may be due to the presence of myocardial stunning areas peripheral to the infarct zone [31, 32]. In this sense, it has been well shown that the presence of a certain degree of post-ischemic dysfunction (stunned myocardium) reverts approximately after 48/72 hours of reperfusion; therefore the change in infarct size in acute experiments does not influence significantly the ventricular function [32].

In summary, Trx1 overexpression reduced, as expected, infarct size in young mice and this cardioprotection included an increase of Akt and GSK-3β phosphorylation. The lack of protection in middle-aged mice was related to the reduction in Trx1 activity and increase in Trx1 nitration. These data, and the fact that in DN-Trx1 we did not detect a reduction in the infarct size, suggest a cardioprotective role of Trx1 activating Akt and GSK-3β in an acute way.

## MATERIALS AND METHODS

### Animal care

All procedures performed in these studies involving animals were in accordance with the ethical standards of the Animal Care and Research Committee of the University of Buenos Aires (CICUAL UBA # 0037016/2012). FVB mice were housed in ventilated cages with a 12 hours light/dark cycle and controlled temperature (20-22°C), and fed with normal chow and water ad libitum.

### Experimental protocols

Three and twelve month-old male hearts from: a) transgenic mice with cardiac- specific overexpression of Trx1 (generated on an FVB background using the α-myosin heavy chain promoter to achieve cardiac-specific expression), b) DN-Trx1 mice was generated by mutation of 32Cys and 35Cys of hTrx1 to Ser using QuikChange (Stratagene, La Jolla, California, USA) [33]. This redox inactive mutant of Trx1 works as a dominant negative for endogenous Trx1 in mice hearts, and c) its corresponding wild type mice (FVB) were randomized into six groups: 1) wild type young (WT-Y, *n* = 8); 2) Trx1 young (Trx1-Y, *n* = 7); 3) wild type middle-aged (WT-MA, *n* = 7); 4) Trx1 middle-aged (Trx1-MA, *n* = 6); 5) DN-Trx1 young (DN-Trx1-Y, *n* = 8); and 6) DN-Trx1middle-aged (DN-Trx1-MA, *n* = 7). For the middle-aged group, mice should be at least 10 month-old; the upper age limit for the middle-aged group is typically 14-15 month-old [34]. For this reason we decided to use 12 month-old as middle-aged mice.

### Isolated mice heart

In all groups we used isolated mice hearts perfused according to Langendorff technique and performed 30 minutes of global ischemia and 120 minutes of reperfusion. The mice were anesthetized by an intraperitoneal injection of sodium pentobarbital (150mg/Kg) and sodium heparin (500 IU/kg bow, i.p). After ensuring sufficient depth of anesthesia, hearts were excised and the aorta was immediately cannulated with a 21 gauge cannula. After that, hearts were perfused according to Langendorff technique with Krebs bicarbonate-buffered solution containing (in mM): NaCl 118.5, KCl 4.7, NaHCO_3_ 24.8, KH2PO_4_ 1.2, Mg SO_4_ 1.2, CaCl_2_ 1.5 and glucose 10. This solution was continuously bubbled with 95% O_2_ and 5% CO_2_ (pH = 7.4) at 37°C. During this time a small fluid-filled balloon, which was connected *via* a thin plastic catheter (P50) to a pressure transducer (Deltram II, Utah Medical System), was inserted into the left ventricle (LV) *via* the left atrium. The catheter with the transducer was positioned in such a way that it secured the position of the balloon in the left ventricle for measurement of LV pressure. The latex balloon was filled with water to achieve an LV end diastolic pressure of 8-10 mmHg (LVEDP). Two electrodes were sutured and connected to a pacemaker to produce a constant heart rate of 472±30 beats/min. We also recorded the coronary perfusion pressure (CPP) through a pressure transducer connected to the perfusion line. All hearts were perfused at constant flow at 4.01±0.27 ml/min. Coronary flow was adjusted to obtain a CPP of 73±3 mmHg during the initial stabilization period and was then maintained constant throughout the experiment. LVDP and LV+dP/dt_max_ were used as contractile state indexes. LVEDP, a myocardial stiffness index in the isovolumic heart, was also measured.

### Infarct size measurement

The assessment of the infarct size was performed using 2,3,5-triphenyltetrazolium chloride (TTC). After 120 minutes of reperfusion, the hearts were frozen and cut into 1 mm transverse slices from apex to base. Sections were incubated for 20 minutes in 1% TTC (pH 7.4, 37°C) and then immersed in 10% formalin. With this technique, viable sections were stained red, while the non-stained sections corresponded to the infarct area. Sections were traced to acetate sheets and planimetered (Image Pro Plus, version 4.5). Infarct size was expressed as a percentage of the left ventricular area.

### Insulin reduction assay for Trx1

The activity of Trx1 in the heart was determined by the insulin reduction assay, according to the method described by Holmgren and Bjornstedt (1995) with a slight modification. Hearts form TG Trx1 young mice (*n* = 5) and MA (*n* = 6) were homogenated with ice-cold phosphate buffered saline (PBS) containing 10 μg/ml PMSF (phenylmethanesulfonylfluoride), 0.5 μg/ml aprotinin and 0.5 μg/ml leupeptin. An equal amount of protein (50 μg) in a volume of 13.28 μl was preincubated with 3.32 μl of the dithiothreitol (DTT) activation buffer (100 mM Tris-Cl [pH 7.5], 2 mM ethylenediaminetetraacetic acid (EDTA), 1 mg/ml bovine serum albumin (BSA), and 2 mM DTT) at 37°C for 15 minutes. The samples were then mixed with 183.3 μl of reaction mixture (100 mM Tris-Cl [pH 7.5], 2.0 mM EDTA, 1.0 μg human Trx-reductase and 140 μM insulin) and were incubated at 25°C. Reaction is started by adding nicotinamide adenine dinucleotide phosphate (NADPH) (0.2 mM). As a control, the samples were mixed with the reaction mixture without Trx-reductase. Changes in absorbance in the absence of Trx-reductase were subtracted from those in the presence of Trx-reductase [33].

### Western blot

We performed additional experiments to obtain hearts samples (*n* = 5 in each group) in order to Western blot analysis. Heart tissue was homogenized in ice for approximately 2 minutes with extraction buffer (pH 7.4), composed of: Tris 1.2 mM, NaCl 0.36 mM, sodium dodecyl sulfate (SDS) 0.1%, Triton 1%, DTT 0.2 mM, protease and phosphatase inhibitors cocktail (Thermo Scientific) at a rate of 500 μL buffer every 150 mg of tissue using a PRO 200 Scientific INC homogenizer. Subsequently, homogenates were centrifuged at 12000 rpm during 20 minutes at 4°C. The supernatant protein concentration was quantified with Bradford method. After protein quantification 50 μg of each sample were separated by 16% Tricine-SDS-PAGE gels for Trx1 and cardiac nitration, and by 12% SDS-PAGE gels for Akt and GSK-3β. Then the gels were transferred to a polyvinylidene fluoride (PVDF) membrane (Thermo Scientific) that was later blocked with 5% BSA for two hours at room temperature. Subsequently, the membrane was incubated with anti-Trx1 (1:1000) (Cell Signaling), anti-3-nitrotyrosine antibody (1:2000), anti-p-Akt for serine residue 473 and threonine residue 308 (1:1000) (Cell Signaling), and anti-p-GSK-3β for serine residue 9 (1:1000) (Cell Signalling) over night at 4°C with agitation. It was later incubated with anti-rabbit secondary antibody conjugated with horseradish peroxidase (HRP, 1:15000) (Millipore) for an hour at room temperature. The membrane was developed with photographic plates (Kodak) and Super Signal West Pico Chemiluminescent Substrate (Thermo Scientific). Proteins expression was quantified by densitometry with Image Gauge 4.0 software (Fujifilm) compared to the charge control values, Ponceau Red staining (Sigma) measured in the same membranes and was used as loading control.

### Statistical analysis

Data are expressed as mean ± standard error of the mean (SEM). Inter-group comparisons were performed using analysis of variance and then the Bonferroni test for multiple comparisons. *p* < 0.05 was considered statistically significant.
